# miR-429 Inhibits Differentiation and Promotes Proliferation in Porcine Preadipocytes

**DOI:** 10.3390/ijms17122047

**Published:** 2016-12-07

**Authors:** Ying Peng, Fen-Fen Chen, Jing Ge, Jia-Yu Zhu, Xin-E Shi, Xiao Li, Tai-Yong Yu, Gui-Yan Chu, Gong-She Yang

**Affiliations:** 1 Laboratory of Animal Fat Deposition & Muscle Development, College of Animal Science and Technology, Northwest A&F University, Xianyang 712100, China; py9101@163.com (Y.P.); ffchen03@sina.com (F.-F.C.); jingjingdehnaa@163.com (J.G.); jiayuzhulam@163.com (J.-Y.Z.); xineshi@nwsuaf.edu.cn (X.-E.S.); nice.lixiao@gmail.com (X.L.); yutaiyong310@nwsuaf.edu.cn (T.-Y.Y.); yz97721@gmail.com (G.-Y.C.); 2 School of Life Sciences, Southwest Forestry University, Kunming 650224, China

**Keywords:** miR-429, proliferation, differentiation, porcine subcutaneous preadipocytes, porcine intramuscular preadipocytes, KLF9, p27

## Abstract

MicroRNAs (miRNAs) are crucial regulatory molecules for adipogenesis. They contribute to the controlling of proliferation and differentiation of preadipocytes. Previous studies revealed an important role of miR-429 in cell invasion, migration, and apoptosis. Our previous work has shown that the expression of miR-429 in subcutaneous fat can be observed in newly born (3-day-old) Rongchang piglets rather than their adult counterparts (180-day-old). This expression pattern suggests that miR-429 might be functionally related to postnatal adipogenesis. However, we currently lack a mechanistic understanding of miR-429 within the context of preadipocyte differentiation. In this study, we investigated the function of miR-429 in porcine subcutaneous and intramuscular preadipocyte proliferation and differentiation. In our porcine preadipocyte differentiation model, miR-429 expression decreased remarkably upon adipogenic induction. Overexpression of miR-429 notably down-regulated the expression of adipogenic marker genes: *PPARγ*, *aP*2, *FAS* and impaired the triglyceride accumulation, while the expression of lipolytic gene *ATGL* was not affected. In addition, we observed that miR-429 significantly promoted the proliferation of porcine preadipocytes. We also found that miR-429 could directly bind to the 3′-UTRs of *KLF9* and *p27*, which have been well documented to promote preadipocyte differentiation and repress cell cycle progression. Taken together, our data support a novel role of miR-429 in regulating porcine preadipocyte differentiation and proliferation, and *KLF9* and *p27* are potent targets of miR-429 during these processes.

## 1. Introduction

White adipose tissue (WAT) controls body energy homeostasis by storing energy in the form of triglyceride and secreting adipocytokines [1]. For livestock, abnormal fat deposition seriously affect the yield and quality of meat [2]. In contrast, Moderate intramuscular fat deposition can both increase muscle tenderness and improve meat flavor [3]. Therefore, the study on WAT holds the possibility to better control the fat content in stock meat production industry [4]. Based on the distribution within the body, mammalian WAT are mainly categorized into subcutaneous adipose tissue, visceral adipose tissue and intramuscular fat tissue. Research has shown that porcine lipid metabolism and physiological function differ between intramuscular and subcutaneous fat cells [5]. Therefore, it is necessary to study the mechanism of adipogenesis in both cell lines. Here, we used porcine subcutaneous pre-adipocytes (PSPAs) and porcine intramuscular pre-adipocytes (PIPAs) as experimental materials to study the molecular mechanisms of preadipocyte adipogenesis.

MicroRNAs (miRNAs) are defined as endogenous non-coding single-stranded RNAs with the length of ~22-nucleotide. MiRNAs function in cell fate decision, cell proliferation, differentiation, apoptosis and tumorigenesis by binding mRNAs at 3′-UTR region, and this binding causes the cleavage or translational inhibition of the mRNA [6,7]. MiRNAs are also important regulatory molecules for adipogeneis [8,9]. Recently, an increasing number of studies using pigs as models have shed light on the adipogenic function study of miRNAs. Li et al. showed that miRNA-181(miR-181) is a positive regulator of porcine adipogenesis [10], whereas another research discovered that miR-33b, which is embedded in intron 16 of porcine sterol regulatory element binding transcription factor (SREBP), down-regulated preadipocyte differentiation through targeting early B cell factor 1 [11]. Other miRNAs, such as miR-146a-5p, miR-199a-5p, miR-130b, miR-145 and miR-103 are also functionally elucidated in porcine adipogenesis [12,13,14,15,16].

The miR-200 family consisting of five member: miR-200a, miR-200b, miR-200c, miR-429 and miR-141, which are highly conserved among their seed sequence [17]. Previous studies about miR-200s are mainly focused on the regulation of tumor progression and the epithelial-mesenchymal-transition (EMT) [18,19]. Among them, miR-429 plays an important role in a variety of cellular processes, such as cell invasion, migration, proliferation and apoptosis [20,21,22,23,24,25]. Knockout of miR-200b/a/429 caused high-fat-diet-induced obesity and insulin resistance [26]. In addition, Wang et al. showed that the expression of gga-miR-429 was 2.3-fold lower in fat chicken line compared to the lean one [27]. Our previous work has shown that the expression of miR-429 in subcutaneous fat can be observed in newly born (3-day-old) Rongchang piglets rather than their adult counterparts (180-day-old) [28].

In this study, we profiled the expression pattern of miR-429 during in vitro adipogenesis of PSPAs and PIPAs. We found that overexpression of miR-429 inhibited adipogenesis and attenuated the lipid accumulation, which were partially mediated by targeting a positive regulator in adipogenesis [29], *KLF9*. Our data also showed that miR-429 accelerated the proliferation of porcine preadipocytes, and further experimentation validated that miR-429 could target on *p27* that have an inhibitory effect on cell cycle [30]. Taken together, our study suggests that miR-429 is a negative regulator in porcine subcutaneous and intramuscular preadipocyte differentiation.

## 2. Results

### 2.1. miR-429 Is Ubiquitously Expressed in Various Porcine Tissue Types and Downregulated in Early Differentiation of Porcine Preadipocytes

**Figure 1:**
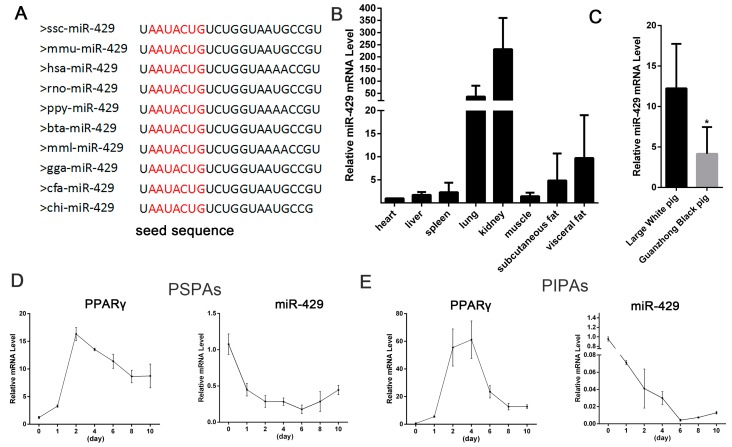
miR-429 tissue distribution and expression patterns during porcine preadipocyte differentiation. (**A**) Mature miR-429 sequence was highly conserved among species; (**B**) miR-429 expression was detected in 8 different tissues of 180-day Large White pig; (**C**) RT-qPCR detected the miR-429 expression in subcutaneous adipose tissue of 180-day Large White and Guanzhong Black pigs. *PPARγ* and miR-429 expression pattern during differentiation of PSPAs (**D**) and PIPAs (**E**). Results are indicated as means ± SEM; *n* = 3; * *p* < 0.05. PSPAs, porcine subcutaneous pre-adipocytes; PIPAs, porcine intramuscular pre-adipocytes.

The mature miR-429 sequence was highly conserved among species, such as pig, human, mouse and rat (Figure 1A). To investigate the expression profile of miR-429 in porcine tissues, total RNAs were extracted from various tissues of 180-day Large White pigs. Real-time qPCR (RT-qPCR) data showed that miR-429 was robustly expressed in these tissues, including subcutaneous adipose tissue and muscle tissue (longissimus dorsi) (Figure 1B). Comparing the expression levels of miR-429 in white adipose tissues of two types of pigs (obese, Guanzhong Black; lean, Large White), we found that the expression level of miR-429 was lower in obese type pig (Figure 1C). In order to study the role of miR-429 in PSPAs and PIPAs, we established porcine preadipocyte differentiation model. RNA was extracted from cultured cells at day 0, 1, 2, 4, 6, 8, 10. The induction of the expression of adipogenic markers, *Peroxisome proliferator-activated receptor gamma* (*PPARγ*), confirmed that PSPAs and PIPAs have been normally differentiated (Figure 1D,E). miR-429 expression decreased remarkably at day 1 and reached the minimum at day 6, and then increased slightly in the late stage of differentiation in both cells types (Figure 1D,E). The dramatic change of the miR-429 level after adipogenic induction suggests a potential role of miR-429 in adipogenesis.

### 2.2. miR-429 Inhibits Differentiation of Porcine Subcutaneous Pre-Adipocytes (PSPAs) and Porcine Intramuscular Pre-Adipocytes (PIPAs)

**Figure 2:**
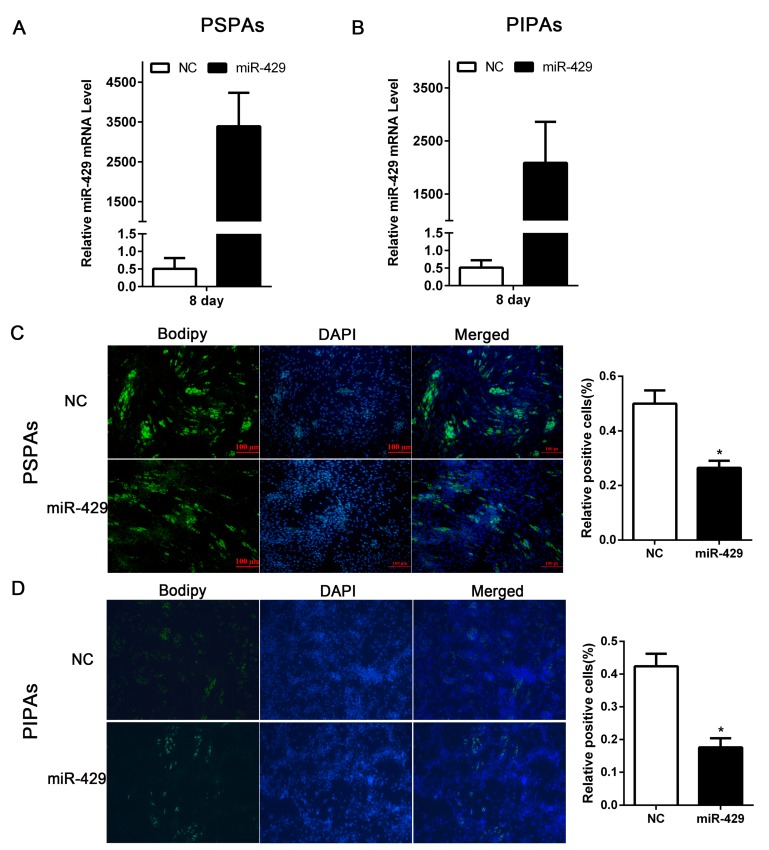
Overexpression of miR-429 inhibit the lipid droplets accumulation in PSPAs and PIPAs. miR-429 agomir or NC was transfected into PSPAs and PIPAs when the density reaching 80%. 24 h later, DMI induce cells differentiation. RT-qPCR was performed on day 8 of adipogenic differentiation to estimate miR-429 overexpression efficiency in (**A**) PSPAs and (**B**) PIPAs. On day 8 of differentiation, PSPAs (**C**) and PIPAs (**D**) cellular lipid droplets were stained with bodipy (green) and cell nuclei were stained with DAPI (blue) (bar size = 100 µm). Statistical results are indicated as means ± SEM; *n* = 3; * *p* < 0.05. NC, negative control; miR-429, miR-429 agomir; DAPI, 4′,6-diamidino-2-phenylindole.

**Figure 3:**
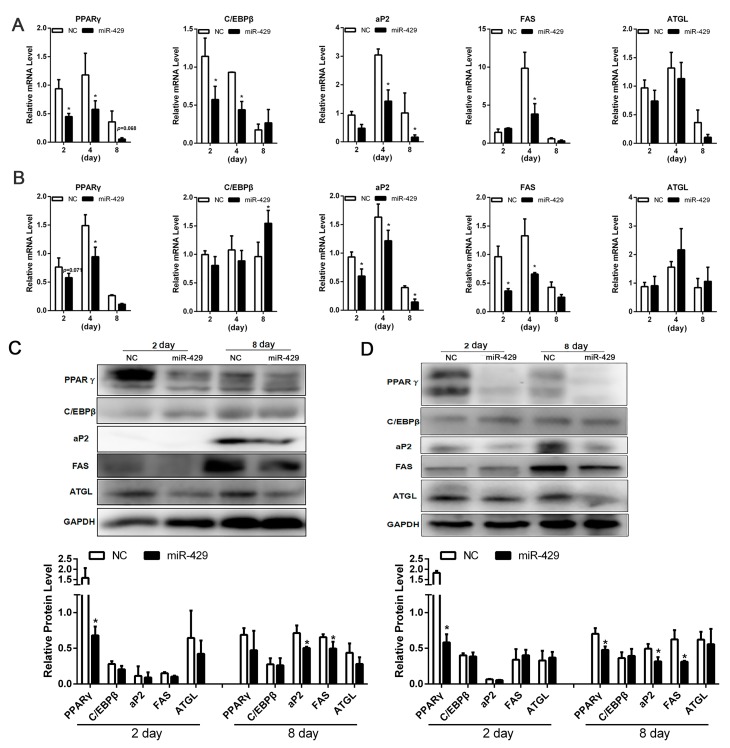
Overexpression of miR-429 inhibit PSPAs and PIPAs differentiation. mRNA expression of *PPARγ*, *aP2, C/EBPβ*, *FAS*, *ATGL* were determined on day 2, 4 and 8 of differentiation. (**A**) was detected in PSPAs; (**B**) was detected in PIPAs. Relative protein level of PPARγ, aP2, FAS, ATGL were detected on day 2 and 8 of differentiation; (**C**) Represented PSPAs; (**D**) Represented PIPAs. Results are indicated as means ± SEM; *n* = 3; * *p* < 0.05.

To elucidate the potential role of miR-429 in adipogenesis, miR-429 agomir or Negative control (NC) was transfected into PSPAs and PIPAs. After 24 h, The adipogenic inducer cocktail DMI (1 µmol/L dexamethasone (DEX), 0.5 mmol/L 3-isobutyl-1-methylxanthine (IBMX), and 5 µg/mL insulin) was used to induce adipogenic differentiation. RT-qPCR showed the overexpression efficiency of miR-429 in both PSPAs and PIPAs at day 8 of differentiation (Figure 2A,B). Lipid droplet staining performed on day 8 of differentiation showed that enforced expression of miR-429 significantly decreased lipid accumulation of porcine subcutaneous adipocytes and intramuscular adipocytes. (Figure 2C,D). Moreover, the mRNA level of adipogenic markers: *PPARγ*, *aP2*, *C/EBPβ* and *FAS* were remarkably reduced in both the miR-429 transfected adipocyte types. However, the expression of lipolytic gene *ATGL* was not affected (Figure 3A,B). Consistently, the protein level of PPARγ, aP2 and FAS were strikingly dampened in treatment group, while C/EBPβ and ATGL did not show significant changes (Figure 3C,D). The above observations suggested that miR-429 has a negative role in porcine adipogenesis.

### 2.3. miR-429 Can Target on KLF9 in PSPAs

**Figure 4:**
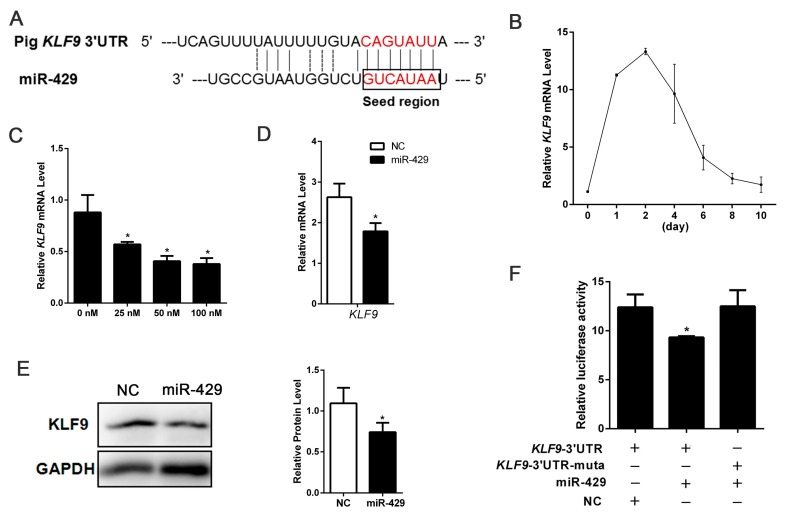
miR-429 can target *KLF9* and downregulate the expression of *KLF9* in PSPAs. (**A**) Target site of miR-429 within porcine *KLF9* mRNA 3′-UTR and mutational site of *KLF9* 3′-UTR, using CGC replace UAU; (**B**) The expression pattern of *KLF9* during porcine subcutaneous preadipocyte differentiation; (**C**) Different concentration miR-429 agomir (0, 25, 50, 100 nM) treated cells, after 48 h of transfection to detect the expression of *KLF9*; The mRNA (**D**) and relative protein (**E**) level of KLF9 were detected on day 2 of PSPAs differentiation; (**F**) *KLF9* mRNA 3′-UTR or its mutation dual-luciferase vector was co-transfected with miR-429 agomir/NC into PSPAs. Dual-luciferase assay was performed at 48 h after transfection. Results are indicated as means ± SEM; *n* = 3; * *p* < 0.05.

To further explore the mechanisms behind the inhibiting effect of miR-429 on porcine adipogenesis, we predicted miR-429 potential targets in computational prediction programs: TargetScan, Pic Tar and miRanda. Among the candidate target genes, we screened potential targets *KLF9* and *ZEB1* which have important role in adpogenesis [29,31]. *KLF9* 3′-UTR was effectively bound to miR-429 seed sequence (Figure 4A). Interestingly, the expression of miR-429 in the differentiation of porcine preadipocytes declined significantly in the early phase and raise up slowly in the late phase, but levels of *KLF9* showed the opposite trend (Figure 1E and Figure 4B). Using different concentrations of miR-429 agomir to treat PSPAs, we found that *KLF9* was inhibited in a dose-dependent manner (Figure 4C). In addition, overexpression of miR-429 remarkably decreased the mRNA and protein expression of KLF9 in PSPAs (Figure 4D,E). However, overexpression of miR-429 had no significant effect on *ZEB1* mRNA and protein levels (Figure S1A,B). Dual Luciferase Reporter Assay showed that co-transfection of miR-429 agomir and *KLF9* 3′-UTR dual-luciferase vector significantly inhibited the dual-luciferase activity (Figure 4F), which directly identified that miR-429 can target *KLF9* in porcine preadipocytes. However, miR-429 agomir has no effect on *ZEB1* 3′-UTR dual-luciferase activity (Figure S1C), indicating that miR-429 does not target the *ZEB1* 3′-UTR in porcine preadipocytes. Taken together, these data showed that miR-429 inhibited the expression of *KLF9* but not *ZEB1* through targeting 3′-UTR in porcine preadipocytes.

### 2.4. miR-429 Promotes Cell Cycle Progression in PSPAs and PIPAs

**Figure 5:**
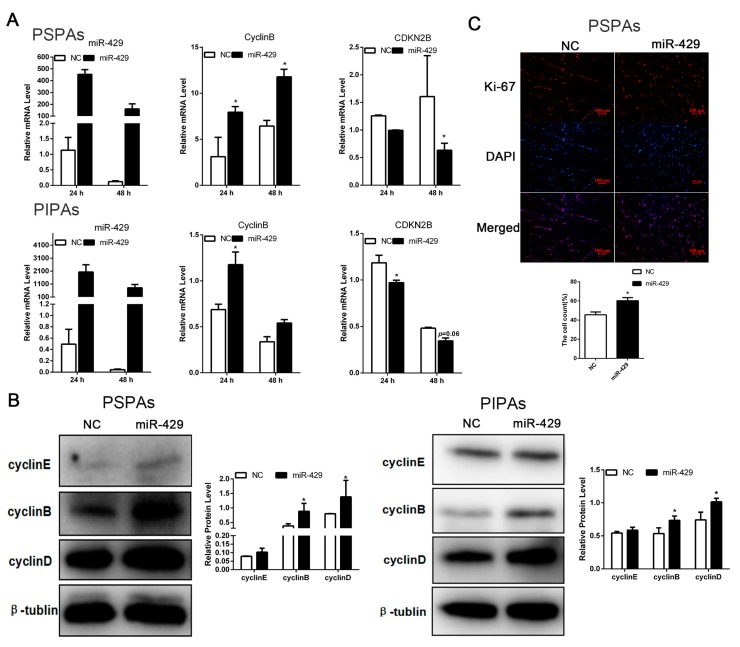
The enhanced miR-429 level upregulated the expression of proliferative marker genes. (**A**) The mRNA expression of miR-429, *Cyclin-B* and *CDKN2B* were detected at 24 and 48 h after transfection in PSPAs and PIPAs; (**B**) Relative protein level of Cyclin E, Cyclin B and Cyclin D were detected by Western Blotting for 48 h of transfection in PSPAs and PIPAs; (**C**) After transfection with miR-429 agomir/NC at 48 h, PSPAs was stained for Ki-67 immunofluorescent staining (bar size = 100 µm). Results are indicated as means ± SEM; *n* = 3; * *p* < 0.05.

**Figure 6:**
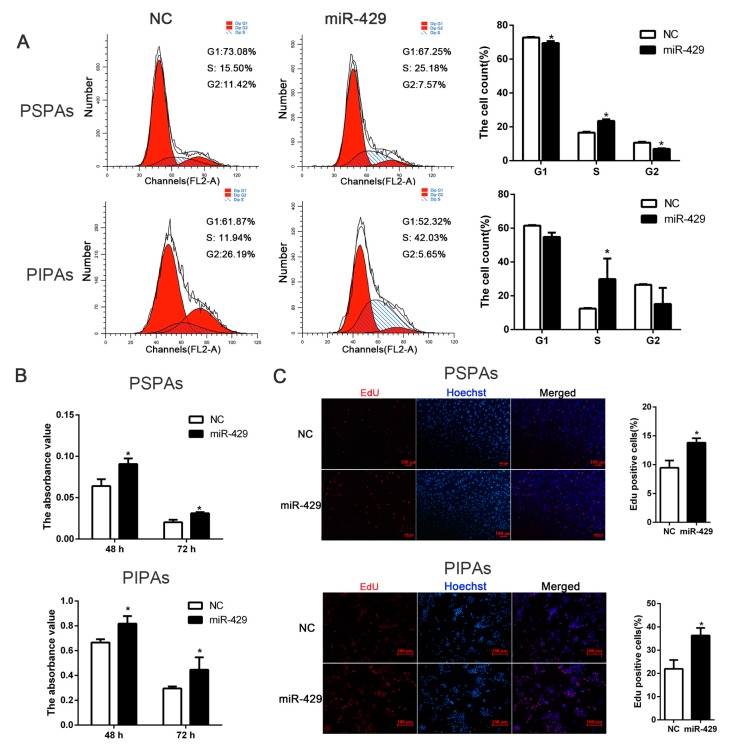
miR-429 significantly enforced proliferation of PSPAs and PIPAs. miR-429 agomir or negative control was transfected into PSPAs and PIPAs when the density reaching 30%. (**A**) Flow cytometry detected cell number of G1, S and G2 phase at 48 h of transfection; (**B**) CCK-8 detected cell vitality at 48 h of transfection; (**C**) EdU staining assayed the number of proliferous cells, positive cells were stained by EdU (red) and total cell nuclei were stained with Hoechst (blue) (bar size = 100 µm). Statistical results are indicated as means ± SEM; *n* = 3; * *p* < 0.05. G1, Gap 1 phase; G2, Gap 2 phase; S, Synthesis phase.

To investigate whether miR-429 also function in the proliferating status of porcine preadipocytes, we set up PSPAs and PIPAs transfectants stably expressing miR-429 using chemical modification agomir. RT-qPCR data showed that miR-429 was successfully overexpressed in both PSPAs and PIPAs, the mRNA level of *Cyclin B* was up-regulated whereas *cyclin-dependent kinase inhibitor 2B* (*CDKN2B*) was down-regulated (Figure 5A). At the protein level, cell cycle related protein Cyclin B and Cyclin D were significantly improved, and Cyclin E also showed a slight increase in both cell types (Figure 5B). Marker of proliferation Ki-67 (Ki-67) is a cellular marker for proliferation, and Ki-67 immunofluorescent staining also reflected the state of the cell proliferation. The result showed that enforced expression of miR-429 promoted the expression of Ki-67 protein (Figure 5C). In order to quantitatively analyze the proportion of cells in different stages of the cell cycle, flow cytometry experiment was performed. As showed in (Figure 6A), ectopic expression of miR-429 notably enhanced the proportion of Synthesis phase (S) cells and dampened the proportion of Gap 1 phase and Gap 2 phase (G1/G2) cells of PSPAs and PIPAs. Moreover, Cell Counting Kit-8(CCK8) detection in both cell types demonstrated that miR-429 enhanced the ability of cell proliferation at 48 and 72 h after transfection (Figure 6B). Consistently, EdU cell proliferation assay indicated that overexpression of miR-429 significantly increased the number of cells in the proliferation period of PSPAs and PIPAs (Figure 6C). Collectively, these results demonstrated that miR-429 could facilitate cell proliferation in PSPAs and PIPAs by regulating the expression of cell cycle related molecules.

### 2.5. miR-429 Is Capable of Targeting p27 in the Proliferation Stage of Porcine Preadipocytes

**Figure 7:**
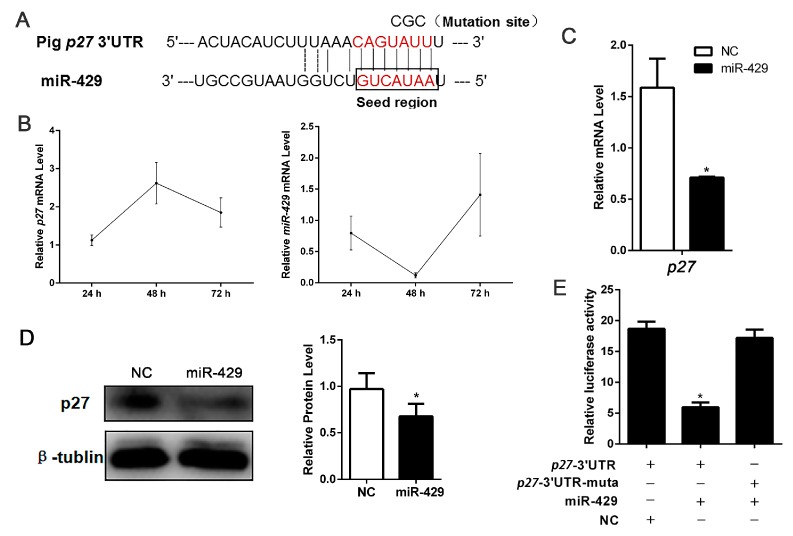
miR-429 can target *p27* and downregulate the expression of p27 in PSPAs. (**A**) Target site of miR-429 within porcine *p27* mRNA 3′-UTR and mutational site of *p27* 3′-UTR (CGC replace UAU); (**B**) miR-429 and *p27* temporal expression at 24, 48 and 72 h; The mRNA (**C**) and relative protein (**D**) level of p27 in PSPAs were detected after 48 h of transfection; (**E**) *p27* mRNA 3′-UTR or its mutation dual-luciferase vector was co-transfected with miR-429 agomir/NC into PSPAs. Dual-luciferase assay was performed at 48 h after transfection. Results are indicated as means ± SEM; *n* = 3; * *p* < 0.05.

To investigate the mechanisms by which miR-429 accelerated cell proliferation, we forecasted and proved the cell cycle related target gene *p27* of miR-429 (Figure 7A). During porcine subcutaneous preadipocyte proliferation, miR-429 was remarkably decreased at 48 h and then increased at 72 h, while *p27* showed the opposite expression pattern (Figure 7B). As predicted, mRNA and protein expression levels of p27 were all inhibited in the miR-429 transfected cell adipocytes (Figure 7C,D). To conclusively validate the target gene *p27*, the *p27*-3′-UTR/the *p27* 3′-UTR mutant dual-luciferase vector were constructed and transfected into PSPAs followed by the transfection with miR-429 agomir/NC. The data indicated that miR-429 markedly suppressed the dual-luciferase activity (Figure 7E). These results suggested that miR-429 could target and inhibit p27 during preadipocyte proliferation.

## 3. Discussion

In this study, we proved that the expression of miR-429 was downregulated during preadipocyte differentiation and the enforced high level of miR-429 blocked adipogenesis. Our data showed that miR-429 was highly conserved among species, suggesting that miR-429 may have a similar function in these species. Consistent with previous data in chicken [27], miR-429 was also lower expressed in the subcutaneous adipose tissue of fat pigs compared with the lean counterparts, implying a role of miR-429 in obesity. Indeed, here we showed that overexpression of miR-429 suppressed adipogenesis in PSPAs and PIPAs. In addition, cell proliferation of PSPAs and PIPAs was accelerated by miR-429 overexpressed. The anti-adipogenic role of miR-429 revealed here was consistent with a recent study where adipocyte-specific miR-200b/a/429 knockout mice was prone to develop high fat diet induced obesity and insulin resistance [26]. Through target gene prediction and validation, our data showed that miR-429 can directly target *KLF9* and *p27* which are crucial.to adipogenesis and cell proliferation respectively. This may contributes to the function of miR-429 in porcine adipocytes. However, we did not observe the down-regulation of miR-429 on a previously reported target gene (Figure S1), *ZEB1* [20,21,31,32]. This may be due to the differences among species and cell lines [33].

Krüppel-like transcriptional factors (KLFs) are important regulators of adipocyte differentiation [34,35,36]. KLF family consists of KLF2–9, KLF13 and KLF15. Among KLF family members, KLF9 is ubiquitously expressed. Previous studies have shown that knockdown of *KLF9* suppressed 3T3-L1 adipocyte differentiation and KLF9, together with C/EBPα was able to transactivate *PPARγ2* expression [29]. Consistently, in HepG2 cells, silencing of *KLF9* inhibited the transcription of *PPARγ* and impaired the lipid accumulation [37]. Further investigation revealed that KLF9 activated the initial phase of 3T3-L1 adipogenesis and suppression of *KLF9* mRNA inhibited adipogenesis through repressing the transcriptional activity of *C/EBPβ* promoter [38]. From these previous studies, it can be seen that suppression of *KLF9* significantly inhibited the differentiation of adipocytes, which were consistent with our data obtained by miR-429 overexpression. In endometrial tissues, *KLF9* was one of target genes of miR-200c during transformation into cancerous states [39]. Due to the seed sequences of miR-200 family members are conservative [17], here we identified that in porcine preadipocytes *KLF9* is also targeted by miR-429 (Figure 4F), indicating that *KLF9* may bind multiple members of miR-200 family. In addition, our result showed that after the overexpression of miR-429, the protein levels of KLF9 and PPARγ were significantly decreased but C/EBPβ was unaffected. Therefore, together with previous studies, we proposed that miR-429 inhibited the expression of PPARγ via targeting *KLF9* in adipogenesis.

Previous studies have testified that miR-429 displayed inconsistent effects on the proliferation of different cell types. Ouyang et al. showed that miR-429 promoted cell proliferation through targeting *p27Kip1* in human prostate cancer cells [23]. While other studies indicated that miR-429 could suppress cell proliferation via down-regulating *BMI1*, *E2F3* and *NOTCH1* expression [22,24]. The discrepancy of the function of miR-429 in different cell lines may due to the diversity of its targets. In our study, we observed that overexpression of miR-429 promoted cell cycle and dampened the expression of *p27* in porcine preadipocytes, which was in line with the study from Ouyang et al. *p27*, a target gene of miR-429, is an important member of KIP family. Research has shown that overexpression of *p27* caused G1 arrest and decreased the cyclin-Cdk activity [40,41]. In addition, Sharma et al. showed that phosphorylated p27 could prevent entry and/or progression of S phase through inhibiting the activity of cyclin E/cyclin A-CDK2 compounds [30]. In our research, our data showed that overexpression of miR-429 dramatically accelerated cell proliferation and enhanced cell number of S phase, which was consistent with the pro-proliferative effect of p27 inhibition. In addition, p27 mRNA and protein expression were significantly inhibited in the miR-429-transfected cells. These data suggested that overexpression of miR-429 accelerated cell proliferation at least partially by targeting *p2*7.

As our cell experimental material, study showed that porcine intramuscular adipocytes have a lower metabolic rate and secrete fewer known adipokines compared to subcutaneous adipocytes [5,42]. Some sequencing data showed that some genes were differentially expressed between porcine intramuscular and subcutaneous preadipocytes and porcine intramuscular adipocytes have less lipid accumulation than subcutaneous adipocytes do at the same differentiation stages [43,44]. In addition, Zhang et al. indicated that porcine subcutaneous preadipocytes grow faster than intramuscular preadipocytes and have higher expression of lipogenic genes [45]. In line with previous studies, we observed that porcine subcutaneous adipocytes have higher ability of differentiation and larger lipid drop size relative to intramuscular adipocytes (Figure 2C,D). Meanwhile, the protein expression of adipogenic genes such as *PPARγ* in PSPAs was higher than that in PIPAs (Figure 3C,D), indicating that there are certain differences of adipogenesis between the two cell lines. Resulting the differences of adipogenic capacity in two kinds of cells may be due to the difference expression of genes, non-coding RNA or microenvironment. Guo et al. found that many miRNAs were differentially expressed between intramuscular and subcutaneous vascular stem cells [9]. Currently, little attention has been allocated to explore whether the same miRNAs play different roles in the two adipocyte types. In this study, we explored the role of miR-429 and observed that miR-429 had the same role in PSPAs and PIPAs differentiation where it negatively regulates the preadipocyte adipogenesis. Therefore, miR-429 may serve as a novel target for reducing pig backfat thickness while increasing lean meat. This is specifically meaningful for Chinese domestic pig breeds which have high backfat thickness and low lean meat content. Because of physiological and anatomical similarities of pigs with human and the similarity adipocyte lipogenic patterns with that of human [46], miR-429 may also have a role in human obesity, and further studies on the function of miR-429 should also be explored in human precursor adipocytes.

## 4. Materials and Methods

### 4.1. Isolation and Culture of PSPAs and PIPAs

PSPAs and PIPAs were respectively isolated from cervical subcutaneous adipose tissue and longissimus dorsi of 3–7 day-old Large White piglets under sterile conditions. Isolated tissues rinsed with phosphate-buffered saline (PBS) three times and then into small pieces, approximate 1 mm^3^. subcutaneous adipose tissue was digested with an equal volume of 1 mg/mL collagenase type I (Invitrogen, Carlsbad, CA, USA) at 37 °C for 45–60 min and longissimus dorsi was digested with an equal volume of 2 mg/mL collagenase type II at 37 °C for 1.5–2 h. 70 µm and 200 µm nylon meshes were utilized to isolate digested cells. The filtrated cell suspension was centrifugation at 1500 rpm for 10 min and then discard the supernatant. Pellets were resuspended and washed two times with DMEM/F12 (Gibco, Grand Island, NY, USA) without fetal bovine serum, then plated cells in growth medium (DMEM/F12) supplemented with 10% fetal bovine serum (Gibco), 100 U/mL penicillin and 100 mg/mL streptomycin. For adipogenic differentiation, when the cell reach confluence, the adipogenic inducer cocktail DMI was added into the growth medium. After 2 days, the medium was changed into the growth medium supplemented with 5 µg/mL insulin for 6–8 days until cell maturation.

Operation of isolating cells from live animals was monitored and demonstrated by the Animal Care Commission of the College of Veterinary Medicine, Northwest A&F University (14-233, 10 December 2014). Porcine sample handling accorded with the ethics committee of Northwest A&F University.

### 4.2. Transfection of miRNA Agomir

miR-429 agomir and NC were purchased from Genepharma (Shanghai, China) and were transfected into the cells by X-tremeGENE HP DNA Transfection Reagent (Roche, Mannheim, Germany). The volume ratio between X-tremeGENE HP DNA Transfection Reagent and miRNA agomir is 1:1. After 24 h transfection, the medium was replaced by growth medium DMEM/F12. The final concentration of miR-429 agomir and NC were 50 nM. The sequence of miRNAs used in this study is as follows:

Ssc-miR-429 sense: 5′-UAAUACUGUCUGGUAAUGCCGU-3′;
Antisense: 5′-GGCAUUACCAGACAGUAUUAUU-3′;
NC sense: 5′-UUCUCCGAACGUGUCACGUTT-3′;
Antisense: 5′-ACGUGACACGUUCGGAGAATT-3′.

### 4.3. RNA Extractions and RT-qPCR

Using Trizol reagent (TaKaRa, Otsu, Japan) extracted the total cellular RNAs according to the manufacturer’s protocol. 500 ng of the total RNA was reversed into cDNA using the Prime Script RT reagent kit (TaKaRa). Quantitative PCR analyses were performed using SYBR green (Vazyme, Nanjing, China) with a BioRad iQ5 system (Bio-Rad, Hercules, CA, USA). Primer sequences used for RT-qPCR analyses were listed in Table 1.

### 4.4. Western Blot Analysis

Cells were split by radio immunoprecipitation assay (RIPA) buffer (Beyotime, Shanghai, China) add with protease inhibitor (Pierce, Rockford, IL, USA). After centrifugation removal cell debris, add 1/4 volume of lysis buffer to the lysates, and then boiling 10 min in water. The total protein sample was pointed into and separated in the SDS-polyacrylamide gel. Then transferred it into a PVDF membrane (Millipore, Boston, MA, USA). Next, the membrane was blocked in 5% defatted milk for 2 h. After that, the membrane was incubated with primary antibodies at 4 °C overnight followed by a secondary antibody at room temperature for 1.5 h. Protein bands were exposure by chemiluminescence reagents (Millipore) and quantified using the Image Lab Image Document. Antibodies PPARγ, ATGL (Cell Signaling, Boston, MA, USA), KLF9 (Abcom, Cambridge, UK), aP2, FAS, cyclin B, cyclin D, cyclin E, p27, ZEB1 (Santa Cruz, Dallas, TX, USA), GAPDH (Boster, Wuhan, China), β-tublin (Sungene Biotech, Tianjin, China) were used.

### 4.5. Luciferase Reporter Assay

The 3′-UTRs of porcine *KLF9* and *p27* containing miR-429 targeted sites were cloned from porcine adipocytes cDNAs using primers tagged with XhoI and NotI (TaKaRa) cutting sites. The wild-type or mutated 3′-UTR fragment was cloned into psiCHECKTM-2 Vector (Promega, Madison, WI, USA) at the 3′-end of the Renilla gene. The structured 3′-UTR dual-luciferase vectors/the 3′-UTR point mutations at positions 3-5 of the *KLF9/p27* seed region dual-luciferase vectors and miR-429agomir/NC were co-transfected into porcine preadipocytes using X-tremeGENE HP DNA Transfection Reagent. Cells were harvested at 48 h post-transfection and assayed basing on the manufacturer’s instructions (Promega).

### 4.6. Bodipy Staining of Lipid Droplets

Differentiated adipocytes of day 8 were washed with PBS three times and fixed with 4% paraformaldehyde at 37 °C for 40 min. Next, cells were washed with PBS three times and incubated with bodipy (Invitrogen) (stock concentration 1 mg/mL, working solution 1:1000 dilution) that is a lipophilic bright green fluorescent dye, for 30 min. The cells were washed three times with PBS and stained nuclei with DAPI (Invitrogen) for 15 min. PBS three times, finally images were captured using a fluorescence microscope (Nikon, Tokyo, Japan).

### 4.7. Flow Cytometry

Porcine preadipocytes were seeded in 5-cm dishes (1.6 × 10^5^ cells per dish). Seeding 24 h later, miR-429 agomir/NC was transfected into porcine preadipocytes. After transfection 48 h, washed porcine preadipocytes three times in PBS and harvested by trypsin digestion. Cells were resuspended in cold 70% ethanol fixing overnight at 4 °C, and then stained with 20 mg/mL propidium iodide (PI) for 30 min. Finally, Flow cytometry instrument (Becton Dickinson, Franklin Lakes, NJ, USA) analysis the cells of cell-cycle.

### 4.8. Cell Counting Kit-8

Porcine preadipocytes were seeded in 96-well plates (2000 cells per dish). After transfection agomir/NC 48 h, CCK8 reagent (Vazyme) were added of 10 µL per dish (lucifugal operation) and then incubate 2–4 h at 37 °C in cell incubator. After that measured the absorbance at 450 nm.

### 4.9. EdU Staining

After transfection 48 h, porcine preadipocytes seeded in 96-well plates (2000 cells per dish) were harvested. Using Cell-LightTM EdU Apollo 567 In Vitro Kit (RIBBIO, Guangzhou, China) detected DNA synthesis. Firstly, added EdU Kit reagent A 50 µmol (dilution for 1:1000) and incubated cells for 2 h at 37 °C in cell incubator. Cells were then fixed in 4% paraformaldehyde for 30 min and neutralize with 2 mg/mL glycine for 5 min. 0.5% Trixon-100 permeabilizing cells with 10 min. According to the instruction manual configuration EdU Reagent B, C, D, E mixture and using mixture incubate cell for 30 min at 37 °C lucifugal operation. Then cells were rinsed with 0.5% Trixon-100 2–3 times and washed with methanol 1–2 times. Hoechst dying nucleus for 30 min and then washed three times in PBS. Images were captured using a fluorescence microscope (Nikon).

### 4.10. Immunofluorescent Staining

Forty-eight hour after transfection, cells were washed with PBS and fixed with 4% paraformaldehyde for 30 min. 0.5% Trixon-100 permeabilizing cells with 10 min. Cells were then blocked with 2% albumin from bovine serum (BSA). After that, cells were incubated with the anti-ki-67 (Novus, NB500-170SS, Littleton, CO, USA) primary antibody (diluted in 2% BSA) at 37 °C for 1.5 h, and fluorescent secondary antibodies incubated cells at 37 °C for 1 h. DAPI stained nuclei for 10 min. Images were captured using a fluorescence microscope (Nikon).

### 4.11. Statistical Analysis

Each experiment was performed three times independently. All quantitative results are represented as mean ± SEM. GraphPad Prism 6 was utilized to graph. One-way analysis of variance (ANOVA) in SPSS 18 software (SPSS Inc., Chicago, IL, USA) was used to perform variance analysis and significance test. * *p* < 0.05.

## 5. Conclusions

**Figure 8:**
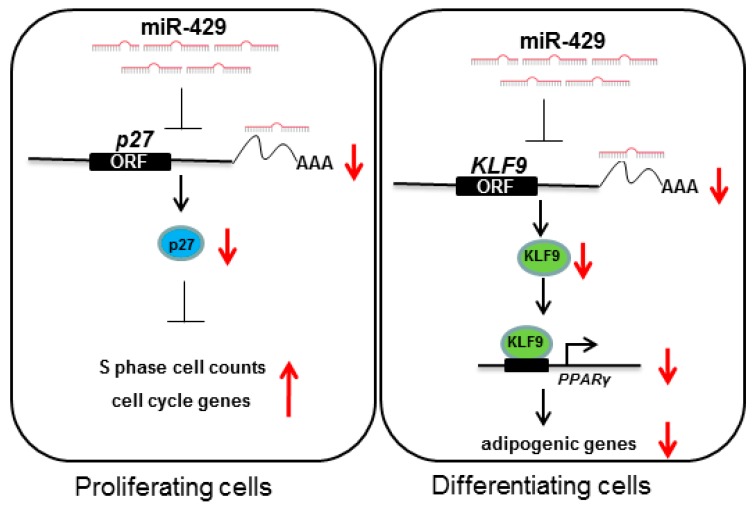
Schematic diagram of cellular mechanism of porcine preadipocytes by miR-429. During porcine preadipocyte proliferation, miR-429 can target on *p27* 3′-UTR to inhibit its expression, then the decreased p27 promote entry and/or progression of S phase [30] and increase the expression of cell cycle genes; During porcine preadipocyte differentiation, miR-429 can target on *KLF9* 3′-UTR to suppress its expression. The reduced KLF9 might inhibit the transcription activity of PPARγ promoter [29]. Then the expression of downstream adipogenic genes were restrained. Red upward arrow means this process is promoted; red downward arrow means this process is inhibited.

In summary, as shown in (Figure 8), overexpression of miR-429 resulted in cell cycle acceleration may partially through targeting *p27* to promote expression of cell cycle genes in proliferating cells. In differentiating cells, miR-429 resulted in lipid droplets reduction may partially via targeting *KLF9* to suppress adipogenic genes. Therefore, miR-429 can function as a negative regulator of adipogenesis in PAPAs and PIPAs. Given the complexity of the miRNAs–target gene network, further investigation may reveal other functional targets of miR-429 in adipogenesis.

## Figures and Tables

**Table 1:** Primer sequences used in this study.

Gene Name	Forward (5′–3′)	Reverse (5′–3′)
*PPARγ*	AGGACTACCAAAGTGCCATCAAA	GAGGCTTTATCCCCACAGACAC
*aP2*	GAGCACCATAACCTTAGATGGA	AAATTCTGGTAGCCGTGACA
*C/EBPβ*	GCACAGCGACGAGTACAAGA	TATGCTGCGTCTCCAGGTTG
*FAS*	CCCCGAATCTGCACTACCAC	AGTTGGGCTGAAGGATGACG
*ATGL*	CCTCATTCCACCTGCTCTCC	GTGATGGTGCTCTTGAGTTCGT
*cyclinB*	AATCCCTTCTTGTGGTTA	CTTAGATGTGGCATACTTG
*CDKN2B*	AGTGGCGGCGGTGGAGAT	GGGTGAGGGTGGCAGGGT
*KLF9*	CGAATCTGGGTCGAGTCCTT	GGGCTTTGAGATGGGAGGAT
*p27*	GGAGGAAGATGTCAAACGTGAG	TCTGCAGTGCTTCTCCAAGTC
*GAPDH*	AGGTCGGAGTGAACGGATTTG	ACCATGTAGTGGAGGTCAATGAAG

## Supplementary Materials

Supplementary materials can be found at www.mdpi.com/1422-0067/17/12/2047/s1.

## Abbreviations

PSPAs	Porcine subcutaneous pre-adipocytes
PIPAs	Porcine intramuscular pre-adipocytes
PPARγ	Peroxisome proliferator activated receptor γ
aP2	Adipocyte fatty acid-binding protein 4
FAS	Fatty acid sythetase
ATGL	Adipose triglyceride lipase
Ki-67	Marker of proliferation Ki-67
Cyclin E	Cell cycle protein E
Cyclin B	Cell cycle protein B
Cyclin D	Cell cycle protein D
KLF9	Krüppel-like transcription factor 9
p27	Cyclin-dependent kinase inhibitor 1B
ssc	*Sus scrofa*
mmu	*Mus musculus*
hsa	*Homo sapiens*
rno	*Rattus norvegicus*
ppy	*Pongo pygmaeus*
bta	*Bos taurus*
mml	*Macaca mulatta*
gga	*Gallus gallus*
cfa	*Canis familiaris*
