# Supplementary material for: miR-429 Inhibits Differentiation and Promotes Proliferation in Porcine Preadipocytes
Source: Int J Mol Sci. 2016 Dec 7;17(12):2047. doi: 10.3390/ijms17122047 (PMC5187847; doi:10.3390/ijms17122047)
Supplement: Supplementary file 1 [file ijms-17-02047-s001.pdf]

Supplementary Material: miR-429 Inhibits Differentiation and Promotes Proliferation in Porcine Preadipocytes

Ying Peng, Fen-Fen Chen, Jing Ge, Jia-Yu Zhu, Xin-E Shi, Xiao Li, Tai-Yong Yu, Gui-Yan Chu and Gong-She Yang

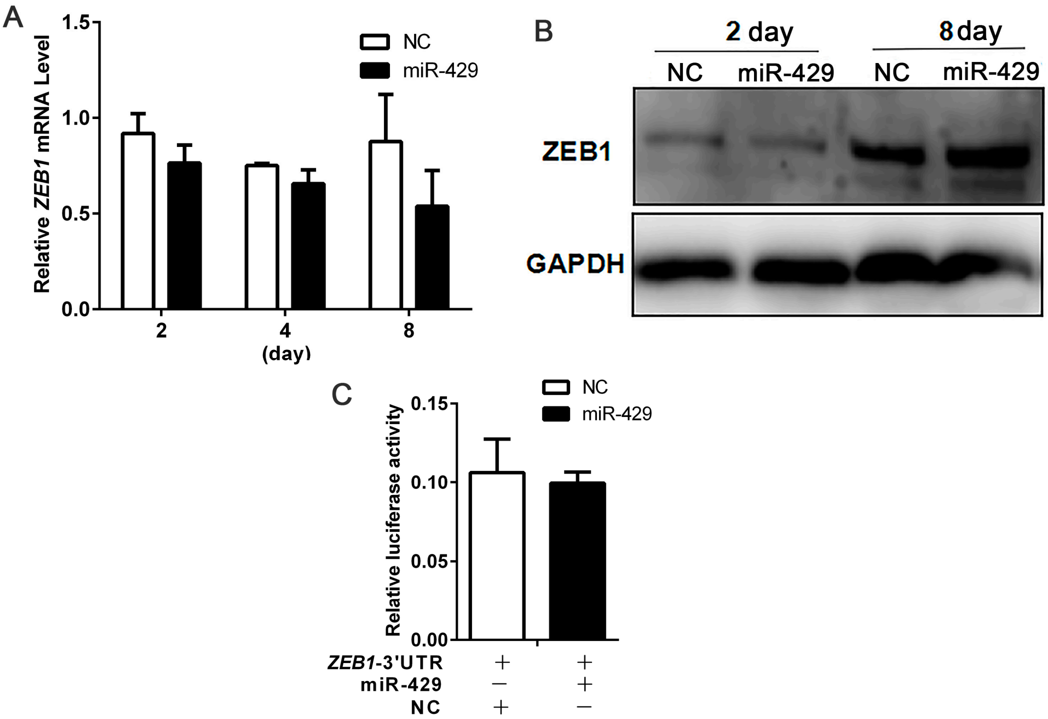

Figure S1. MiR-429 cannot target *ZEB1* and change the expression of ZEB1 in porcine preadipocytes. (**A**) The mRNA and (**B**) relative protein level of ZEB1 in porcine subcutaneous preadipocytes were detect after transfection miR-429 agomir/negative control (NC); (**C**) *ZEB1* mRNA 3′UTR dual-luciferase reporter vectors was co-transfected with miR-429 agomir/NC into porcine subcutaneous preadipocytes. Dual-luciferase assay was performed at 48 h after transfection. Results are indicated as means ± S.D; $n = 3$.
